# Effect of Tranexamic Acid on Blood Management during a High Tibial Osteotomy: A Systematic Review and Meta‐analysis

**DOI:** 10.1111/os.13407

**Published:** 2022-08-01

**Authors:** Qian Fang, Zhen Zhang, Dong Wang, Limin Wang, Wei Xiong, Yunfeng Tang, Wenzheng Liu, Guanglin Wang

**Affiliations:** ^1^ West China Hospital Sichuan University Chengdu China

**Keywords:** Blood management, High tibial osteotomy, Meta‐analysis, Tranexamic acid

## Abstract

This study aimed to evaluate the efficiency and safety of tranexamic acid for blood management during high tibial osteotomy (HTO). A systematic search was conducted in Medline, Embase, and the Cochrane library database. Six studies and 208 patients were included in this meta‐analysis using Review Manager V.5.3 and Stata 15.1 software. For primary outcomes, tranexamic acid lowered the total blood loss (WMD = –219.47, 95% CI [−355.61, −83.33], *P =* 0.002). For secondary outcomes, a significant reduction was found for decreased hemoglobin (POD1: WMD = –9.86, 95% CI [−13.45, −6.28], *P <* 0.05; POD2: WMD = –8.41, 95% CI [−11.50, −5.32], *P <* 0.05; POD5: WMD = –11.48, 95% CI [−14.56, −8.39], *P <* 0.05) and drainage (total: WMD = –105.93, 95% CI [−187.08, −24.78], *P <* 0.05; POD1: WMD = –122.195, 95% CI [−168.902, −75.488], *P <* 0.05). The sex difference (male/female ratio) was determined (total blood loss: *P =* 0.025; total drainage amount: *p =* 0.018) using meta‐regression analysis. Females benefited more from tranexamic acid in terms of total blood loss (M/F > 40%: WMD = –53.11, 95% CI [−100.16, −6.05], *P =* 0.03; 40% ≥ M/F ≥ 20%: WMD = –362.20, 95% CI [−423.96, −300.45], *P <* 0.05; M/F < 20%: WMD = –263.00, 95% CI [−277.17, −248.83], *P <* 0.05) and total drainage (M/F > 40%: WMD = –7.11, 95% CI [−10.75, −3.47], *P <* 0.05; 40% ≥ M/F ≥ 20%: WMD = –104.72, 95% CI [−155.36, −54.08], *P <* 0.05; M/F < 20%: WMD = –222.00, 95% CI [−297.42, −146.58], *P <* 0.05). No significant differences were found for drainage on POD2 and POD3, wound complications, orthromboembolic events. In conclusion, tranexamic acid is effective and safe for blood management during HTO. Females appeared to benefit more from it, and an additional postoperative dose is suggested fora better effect.

## Graphical abstract

Efficiency of tranexamic was showed in reducing blood loss, hemoglobin decreased and drainage in high tibial osteotomy. And safety of it was also presented. Females seemed to benefit more from tranexamic acid and extra postoperative dose was suggested to enhance its effect.

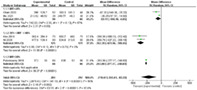

## Introduction

Tranexamic acid (TXA) is an efficient strategy for blood management in surgery, as demonstrated in multiple studies.^1^, ^2^, ^3^ Significant benefits of TXA on blood management have been reported in other types of knee surgery, including arthroplasty and arthroscopic surgery.^4^, ^5^ Compared to other blood management methods, including tourniquet, autologous transfusion, and allogenic transfusion, TXA is relatively cheaper and easier to use. As a synthetic lysine analogue, TXA can competitively bind to lysine‐binding sites on plasminogen to prevent subsequent activation of plasminogen and reduce the fibrinolytic susceptibility of the resulting clot, reducing blood loss.^6^, ^7^ Despite the efficiency of TXA, its safety should also be investigated with respect to possible thromboembolic events, including deep vein thrombosis or pulmonary embolism.^8^

High tibial osteotomy (HTO) is an efficient and extensively used procedure for the treatment of knee osteoarthritis with a varus deformity in the early stages, especially for patients with high activity requirements.^9^ With biplanar osteotomy and an opening gap created on the tibia, the aim of HTO is to realign the weight‐bearing line and shift it from the arthritic tibiofemoral compartment to the opposite healthy joint in order to decrease the load in the diseased compartment, resulting in reduced pain and delayed progression of osteoarthritis.^10^ It enables the preservation of the joint and exerts a large corrective effect on the mechanical axis, which ensures that the patients can live active and demanding lives postoperatively.^10^ Various studies have reported the benefits of HTO, and no significant differences in the clinical outcomes have been found between HTO and unicompartmental knee arthroplasty.^11^, ^12^, ^13^, ^14^

However, perioperative blood loss is theoretically high during HTO, with bone bleeding from the osteotomy gap.^15^ This can increase the incidence of wound complications, anemia, transfusions, and bone non‐union, affecting patients both physically and mentally. Some studies have reported total blood loss of more than 800 mL,^16^, ^17^ delayed wound healing of 1.9%, and superficial wound infection of 1%.^18^ Thus, strategies for blood loss management during a HTO are necessary, and no consensus has been reached.

Nevertheless, studies on the use of TXA during HTO are limited. Previous reviews^19^, ^20^, ^21^ have uncovered a limited number of outcome parameters and high heterogeneity. In this meta‐analysis, more studies were included, influential factors were evaluated, and subgroup analyses were performed. The purpose of this study was to clarify the efficiency and safety of TXA during HTO with multiple outcome parameters and to help surgeons make better clinical decisions.

## Methods

### Search Strategy

Medline (Pubmed), Embase (Ovid), and the Cochrane Central Register of Controlled Trials (Ovid) were searched for potential articles up to 2 June 2021. There were no language or date limitations during the process. Keywords including “tranexamic acid” and “tibial osteotomy” were used with their medical subject heading terms and Embase subject heading terms for all relevant articles. Details of the strategy are presented in Supporting Information 1.

### Selection Criteria

All potential articles were evaluated and selected for further analysis by two reviewers independently. The criteria for inclusion and exclusion are described below. Inconsistencies were resolved with the involvement of a third reviewer. The entire process was conducted according to the PRISMA guidelines. The inclusion criteria were as follows: patients treated only with HTO; TXA was used as the intervention; comparisons were performed between the intervention group and the placebo group; outcomes related to blood loss were reported; randomized controlled trials (RCTs), cohort studies, and case–control studies. Exclusion criteria were as follows: not a full‐text article; low‐quality studies; unrelated to our topic.

### Data Extraction

All data were extracted from eligible articles by two reviewers independently. Discrepancies were resolved with the involvement of a third reviewer. The collected items were as follows: author, publication year, study design, country, route and dose of TXA, sex, age, body mass index (BMI), blood loss, drainage amount, hemoglobin values, decreased hemoglobin, transfusion events, thromboembolic events, and wound complications.

### Quality Assessment

RCTs were evaluated using the Cochrane Collaboration's Tool on seven items, namely random sequence generation, allocation concealment, blinding of participants and personnel, blinding of outcome assessment, incomplete outcome data, selective reporting, and other biases. The Newcastle–Ottawa Scale (NOS) was used to evaluate the cohort and case–control studies. With nine stars in total, studies that scored less than six stars were considered low quality and excluded. Assessments were performed by two reviewers independently, and inconsistencies were discussed and resolved with a third reviewer.

### Statistical Analysis

Review Manager V.5.3 (The Cochrane Collaboration, Oxford, UK) and Stata 15.1 software (StataCorp, College Station, TX, USA) were used to perform the statistical analyses. Forest plots and funnel plots were generated using Review Manager; while meta‐regression, sensitivity analysis, Egger's test, and Begg's test were conducted using Stata. Funnel plots are presented in Supporting Information 2. The mean difference with the standard deviation for continuous outcomes and the relative risk of dichotomous outcomes were summarized, both with their 95% confidence intervals (CIs). *I* ^2^ > 50% or *P <* 0.1 in Q statistics was considered indicative of the existence of heterogeneity. The random‐effects model was applied when heterogeneity was identified; otherwise, the fixed‐effects model was adopted. Sensitivity analysis was conducted by removing one of the included studies at a time followed by re‐analysis to determine the robustness of the study. *P >* 0.1 in Egger's test and *P >* 0.05 in Begg's test were considered to indicate the existence of publication bias.

## Results

### Search Results and Study Characteristics

**Fig. 1:**
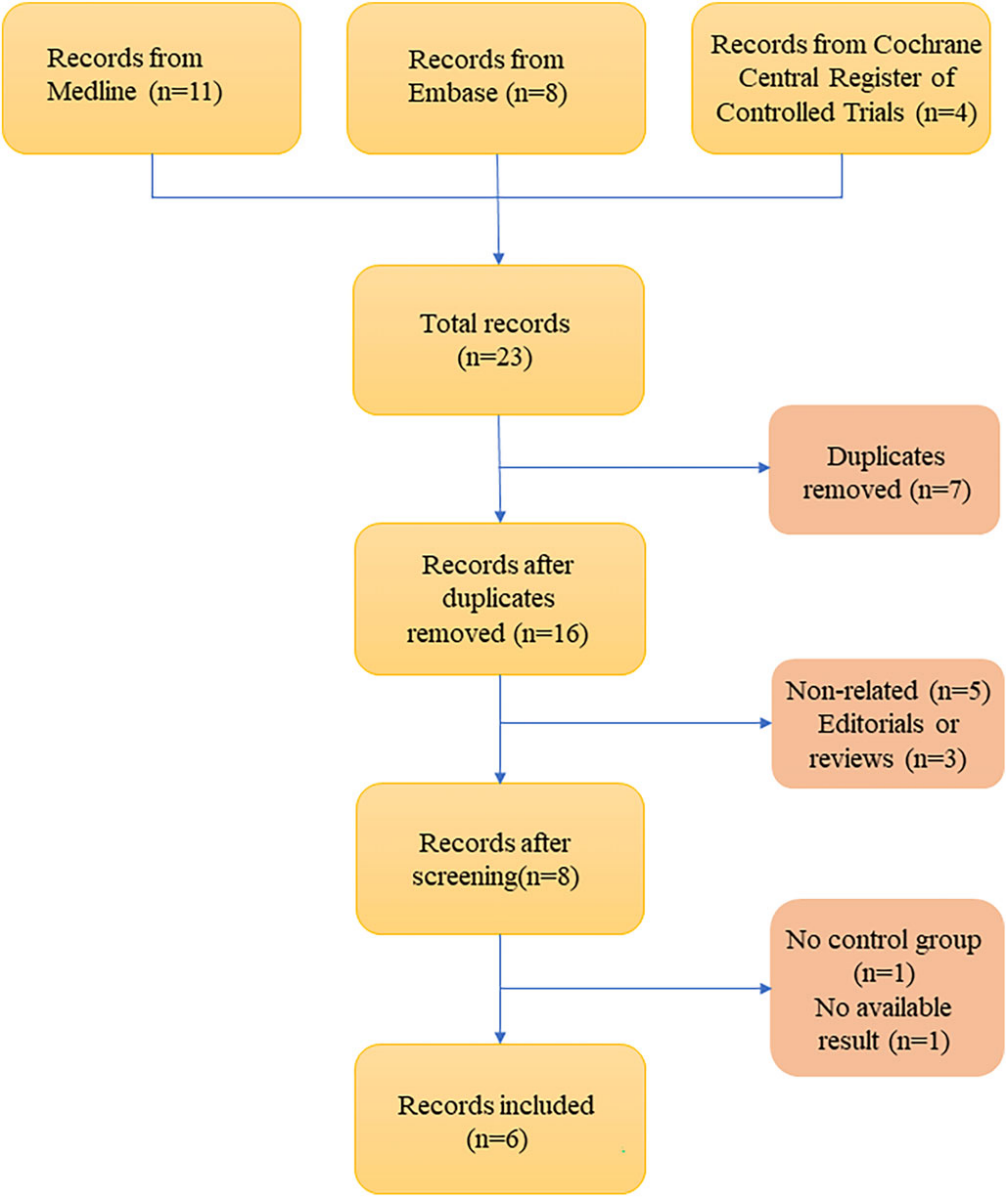
Flow chart for literature screening

A total of 23 articles were collected through a systematic search, among which 11 were from Medline, eight were from Embase, and four were from the Cochrane Central Register of Controlled Trials. Six studies,^16^, ^17^, ^22^, ^23^, ^24^, ^25^ involving 608 patients, were included after screening. The details of the inclusion and exclusion criteria are shown in Fig. Fig. 1.

All six included studies were from Asia, with three from China and three from South Korea. The publication dates ranged from 2018 to 2021. Regarding the study design, two were RCTs, and four were cohort studies. The intravenous route was used in four studies, topical injection was used in one study, and a combination of both was used in one study. All included patients had symptomatic medial compartment knee osteoarthritis with varus deformity requiring correction and underwent an HTO. The age, sex, BMI, and preoperative hemoglobin values of all patients were collected as demographic information. The details are presented in Table 1.

**TABLE 1:** Characteristics of the included studies

Study	Country	Design	Intervention route	Total samples (M/F^†^)	TXA group*				Control group			
					Age (years)	Sex (M/F)	BMI^‡^ (kg/m^2^)	Pre‐Hb^§^ (g/L)	Age (years)	Sex (M/F)	BMI (kg/m^2^)	Pre‐Hb (g/L)
Ma *et al*. (2021)^21^, ^23^	China	RCT**	IV^††^	76 (32/44)	60.78 ± 6.03	14/24	24.19 ± 1.98	112.12 ± 8.34	61.04 ± 5.76	18/20	25.05 ± 1.65	110.98 ± 8.98
Ni *et al*. (2020)^17^	China	RCT	IV	100 (22/78)	52.5 ± 2.8	10/40	23.2 ± 1.5	131 ± 7	52.9 ± 3.1	12/38	23.5 ± 1.3	131 ± 4
Chen *et al*. (2020)^22^	China	Cohort study	IV + topical	100 (42/58)	58.3 ± 10.4	20/32	27.3 ± 4.0	140.0 ± 16.5	56.6 ± 10.2	22/26	28.5 ± 4.2	137.4 ± 15.0
Kim *et al*. (2018)^16^	South Korea	Cohort study	IV	150 (31/119)	55.0 ± 6.8	17/58	26.3 ± 3.1	132 ± 10	55.7 ± 5.5	14/61	26.4 ± 2.6	132 ± 11
Palanisamy *et al*. (2018)^24^	South Korea	Cohort study	IV	152 (16/136)	58 ± 5	7/59	27 ± 2	130 ± 5	57 ± 6	9/77	26 ± 2	130 ± 4
Suh *et al*. (2018)^25^	South Korea	Cohort study	Topical	30 (7/23)	60 ± 5.6	3/12	28.1 ± 3.9	130 ± 10	56 ± 5.7	4/11	26.1 ± 2.7	129 ± 9

*Notes*: *Tranexamic acid.

^†^ Ratio of males to females.

^‡^ Body mass index.

^§^ Preoperative hemoglobin level.

** Randomized controlled trial.

^††^ Intravenous.

### Quality Assessment of Studies

**Fig. 2:**
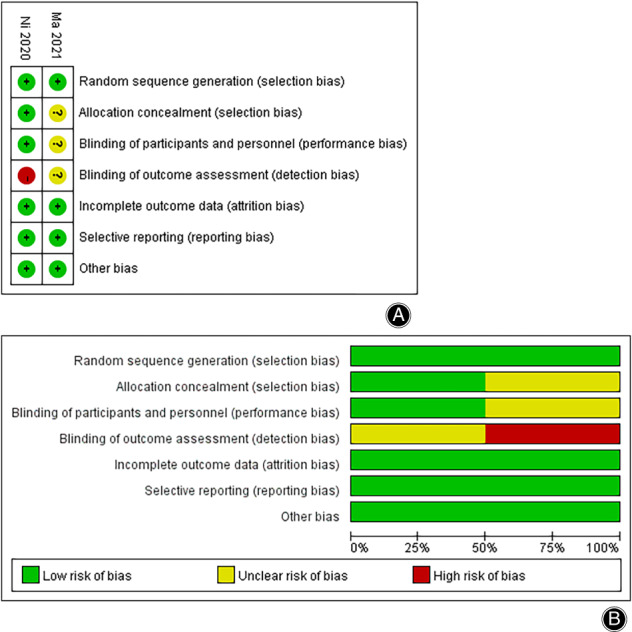
Summary (A) and graph (B) of quality assessment for randomized controlled trials.

Two RCTs were evaluated with Cochrane Collaboration's Tool, and the graphs and summary are presented in Fig. 2. Four cohort studies were evaluated with the Newcastle–Ottawa Scale, among which one had a score of 9, two had a score of 8, and one had a score of 7. The detailed evaluations of the studies are shown in Table 2. All studies were considered to be high quality, and none were excluded.

**TABLE 2:** Quality assessment for the included cohort studies

Newcastle–Ottawa scale item		Chen *et al*. (2020)^22^	Kim *et al*. (2018)^16^	Palanisamy *et al*. (2018)^24^	Suh *et al*. (2018)^25^
Selection	Representativeness of the exposed cohort	1	1	1	1
	Selection of the nonexposed cohort	1	1	1	1
	Ascertainment of exposure	1	1	1	1
	Demonstration that the outcome of interest was not present at the start of the study	1	1	1	1
Comparability	Comparability of cohorts on the basis of the design or analysis	2	2	2	2
Outcome	Assessment of outcome	1	0	1	1
	Follow‐up was long enough for outcomes to occur	1	1	0	1
	Adequacy of follow‐up of cohorts	0	1	0	1
Total score		8	8	7	9

### Primary Outcomes

#### Total Blood Loss

**Fig. 3:**
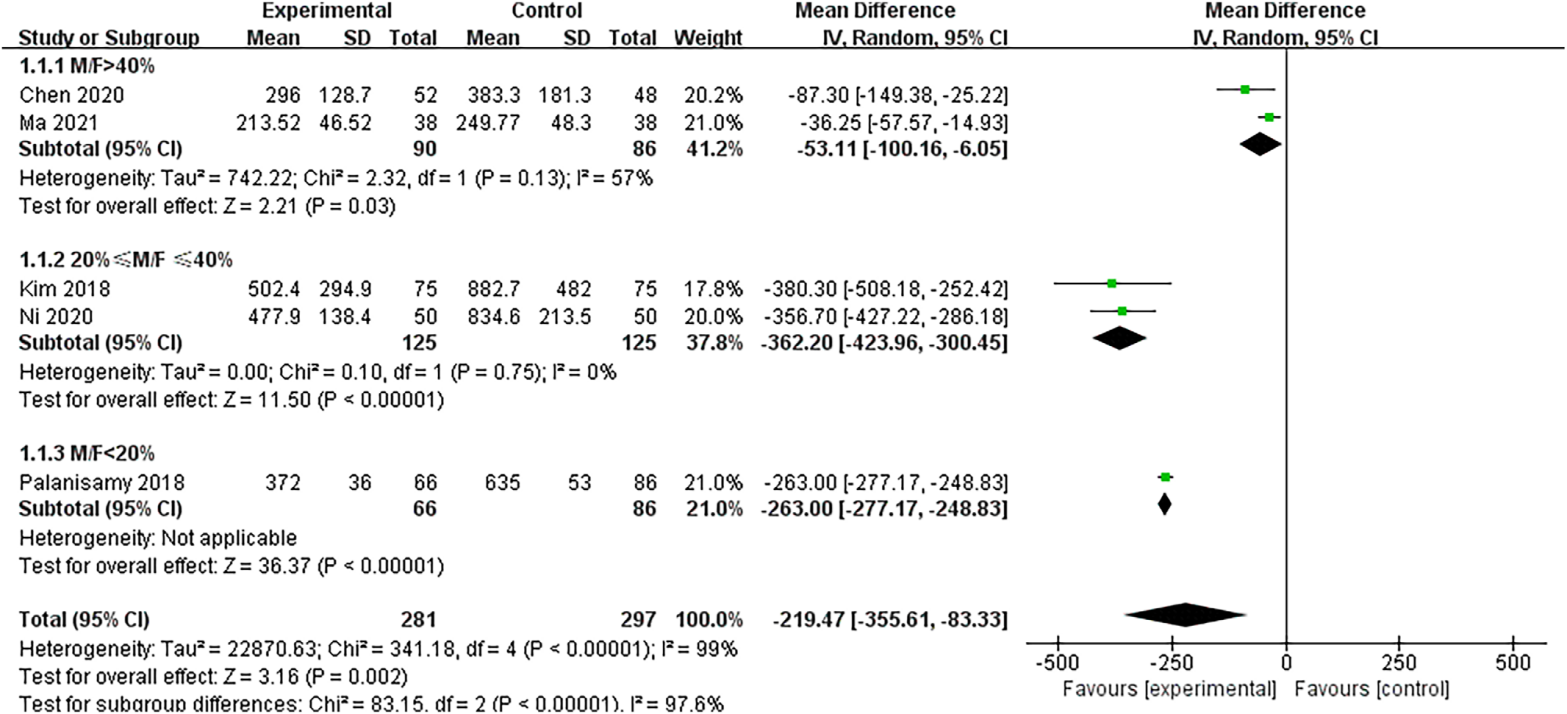
Forest plot of total blood loss during high tibial osteotomy in the TXA and control groups. Pooled results of the overall effect and subgroups are shown. Subgroups were generated based on M/F. Abbreviations: TXA, tranexamic acid; M/F, ratio of males to females

In total, five studies included data on the perioperative blood loss, which was a combination of the apparent and hidden blood loss. However, heterogeneity was extremely high (*I* ^2^ = 99%, *P <* 0.0001 of Q statistics) in all five articles in the random‐effects model. Thus, demographic items were evaluated using meta‐regression analysis, including the total number of patients (*P =* 0.605), average age (*P =* 0.052), average BMI (*P =* 0.575), average preoperative hemoglobin values (*P =* 0.581), and sex (male/female ratio, M/F, *P =* 0.025). Three subgroups were created based on sex: group 1 (M/F > 40%), group 2 (40% ≥ M/F ≥ 20%), and group 3 (M/F < 20%), and a random‐effects model was applied. There were two studies in group 1 (*I* ^2^ = 57.0%, *P =* 0.13 in Q statistics), two studies in group 2 (*I* ^2^ = 0%, *P =* 0.75 in Q statistics), and one study in group 3 (*I* ^2^ and Q statistics were not available). Total blood loss was significantly reduced by TXA administration in all three groups: group 1 (weighted mean difference (WMD) = −53.11, 95% CI [−100.16, −6.05], *P =* 0.03), group 2 (WMD = –362.20, 95% CI [−423.96, −300.45], *P ≤* 0.05), and group 3 (WMD = –263.00, 95% CI [−277.17, −248.83], *P ≤* 0.05). The difference between subgroups was also significant (*P <* 0.05), confirming the necessity of subgroup analysis. The overall effect of blood loss reduction was significant as well (WMD = –219.47, 95% CI [−355.61, −83.33], *P =* 0.002), but the results were suspicious because of high heterogeneity (Fig. 3, Fig. S1).

#### Transfusion

**Fig. 4:**
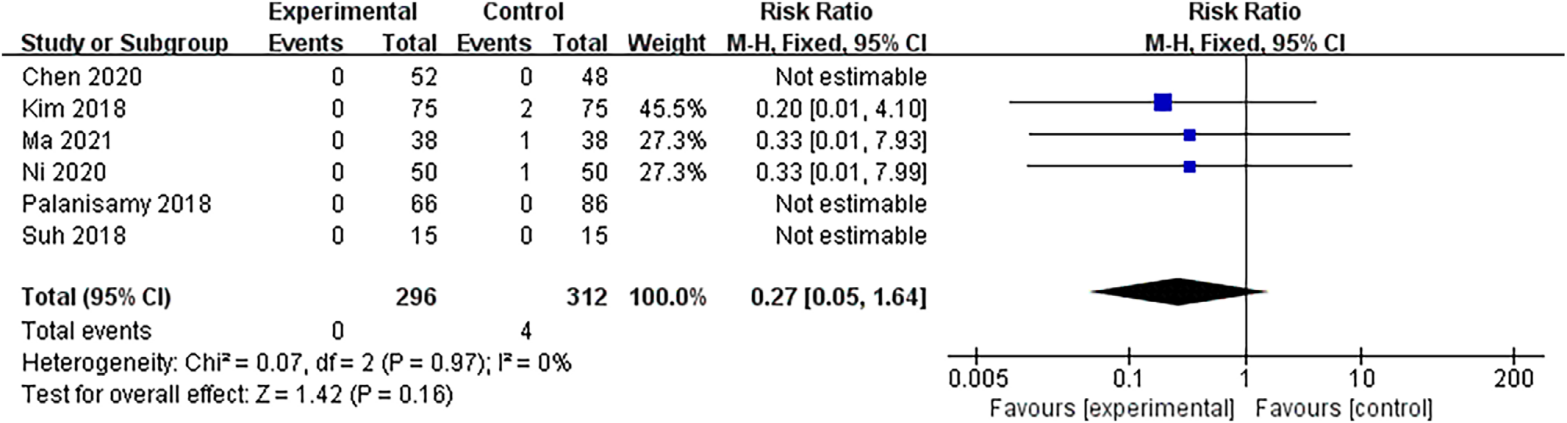
Forest plot of transfusion during a high tibial osteotomy in the TXA and control groups. Abbreviation: TXA, tranexamic acid

Three studies were included in the analysis of transfusion, and nonsignificant heterogeneity was found (*I* ^2^ = 0%, *P =* 0.97 in Q statistics). The fixed‐effects model showed no significant difference between the TXA and non TXA (NTXA) groups (relative risk (RR) = 0.27, 95% CI [0.05, 1.64], *p =* 0.16) (Fig. 4, Fig. S2).

### Secondary Outcomes

#### Decreased Hemoglobin on Postoperative Day 1 (POD1)

**Fig. 5:**
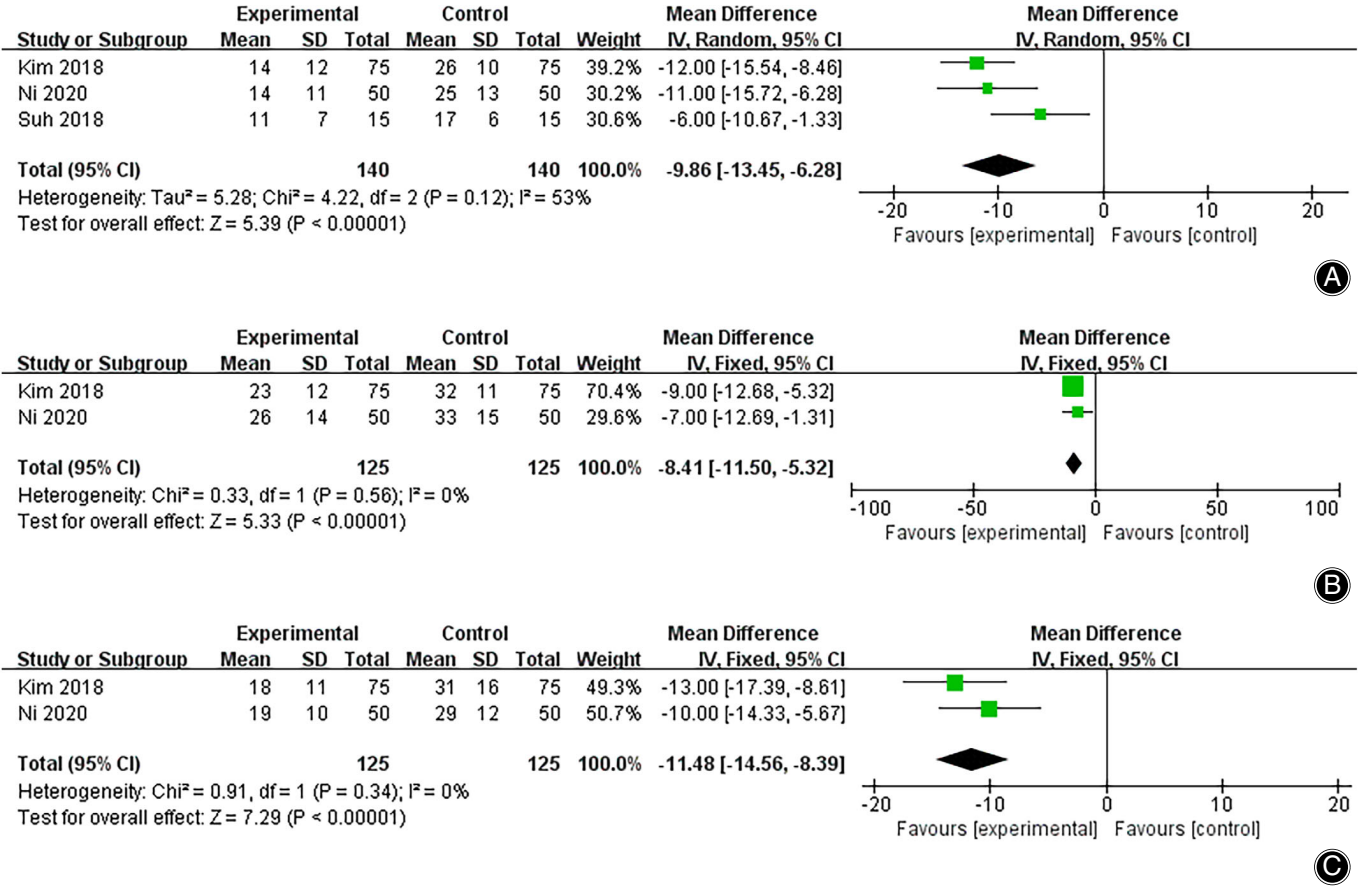
Forest plot of decreased hemoglobin on POD1 (A), POD2 (B), and POD5 (C) after high tibial osteotomy between the TXA and control groups. Abbreviations: POD1, postoperative day 1; POD2, postoperative day 2; POD5, postoperative day 5; TXA, tranexamic acid

The change in the hemoglobin value was calculated by subtracting the preoperative hemoglobin value from the postoperative hemoglobin value. Three studies reported a change in the hemoglobin value on POD1. A random‐effects model was applied (*I* ^2^ = 55%, *P =* 0.12 in Q statistics),and no significant difference was identified using meta‐regression (total number of patients: *P =* 0.334, age: *P =* 0.407, BMI: *P =* 0.559, preoperative hemoglobin value: *P =* 0.296, sex ratio: *P =* 0.257). The TXA group exhibited a significantly smaller change in the hemoglobin value on POD1 compared to the NTXA group (WMD = –9.86, 95% CI [−13.45, −6.28], *P <* 0.05) (Fig. 5A, Fig. S3).

#### Decreased Hemoglobinon Postoperative Day 2 (POD2)

Two studies reported a change in the hemoglobin value on POD2 (*I* ^2^ = 0%, *P =* 0.56 in Q statistics). Analysis with a fixed‐effects model indicated that the change in the hemoglobin value was significantly less in the TXA group compared to that in the NTXA group (WMD = –8.41, 95% CI [−11.50, −5.32], *P <* 0.05) (Fig. 5B, Fig. S4).

#### Decreased Hemoglobinon Postoperative Day 5 (POD5)

A change in the hemoglobin value on POD5 was reported in two studies. The fixed‐effects model (*I* ^2^ = 0%, *P =* 0.34 in Q statistics) indicated that the TXA group exhibited a significantly greater change in the hemoglobin value compared to that of the NTXA group (WMD = –11.48, 95% CI [−14.56, −8.39], *P <* 0.05) (Fig. 5C, Fig. S5).

#### Total Drainage Amount

**Fig. 6:**
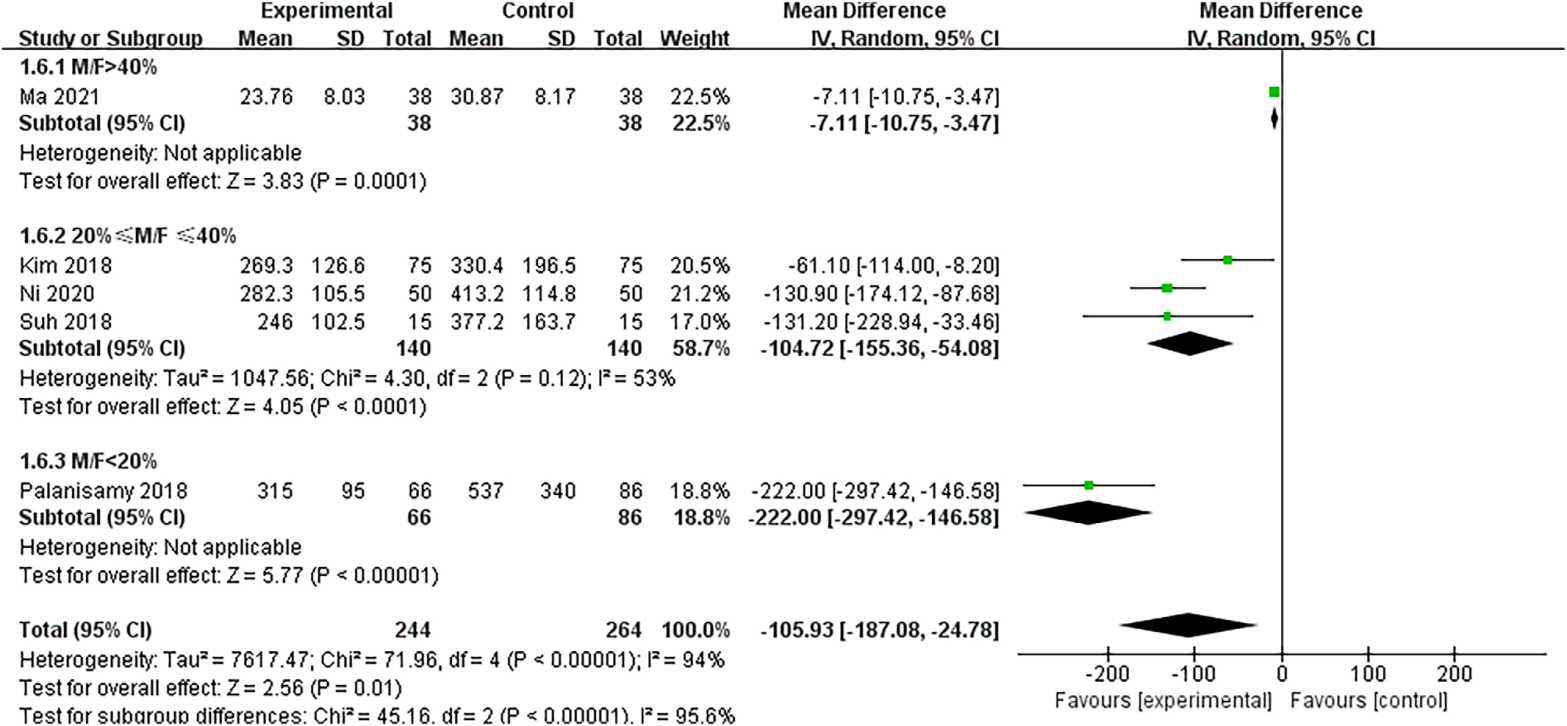
Forest plot of total drainage after a high tibial osteotomy in the TXA and control groups. Pooled results of the overall effect and subgroups are shown. Subgroups were generated based on M/F. Abbreviations: TXA, tranexamic acid; M/F, ratio of males to females

Analysis of five of the included studies showed significant heterogeneity in the total drainage amount (*I* ^2^ = 94%, *P <* 0.0001 in Q statistics). The demographic items were used for meta‐regression analysis, among which sex (M/F ratio) showed significance (*P =* 0.018), unlike the total number of patients (*P =* 0.611), age (*p =* 0.451), BMI (*p =* 0.752), and preoperative hemoglobin value (*P =* 0.260). Subgroups were set according to the sex ratio, with one study in group 1 (M/F > 40%), three studies in group 2 (40% ≥ M/F ≥ 20%),and one study in group 3 (M/F < 20%). A heterogeneity test could not be performed for groups 1 and 3, but heterogeneity was found in group 2 (*I* ^2^ = 53%, *P =* 0.12 in Q statistics). A random‐effects model was applied and indicated significant decreases in the total drainage amount in the TXA group in all three subgroups as follows: group 1 (WMD = –7.11, 95% CI [−10.75, −3.47], *P <* 0.05), group 2 (WMD = –104.72, 95% CI [−155.36, −54.08], *P <* 0.05), and group 3 (WMD = –222.00, 95% CI [−297.42, −146.58], *P <* 0.05). Significant differences were also identified between different subgroups (*P <* 0.05), confirming the necessity of subgroup analysis. Analysis of the overall effect showed significantly less total drainage in the TXA group (*I* ^2^ = 94%, *P <* 0.0001 in Q statistics, WMD = –105.93, 95% CI [−187.08, −24.78], *P <* 0.05) compared to the NTXA group. However, the overall effect should be interpreted cautiously due to high heterogeneity (Figure. 6, Fig. S6).

#### Drainage Amount on POD1

**Fig. 7:**
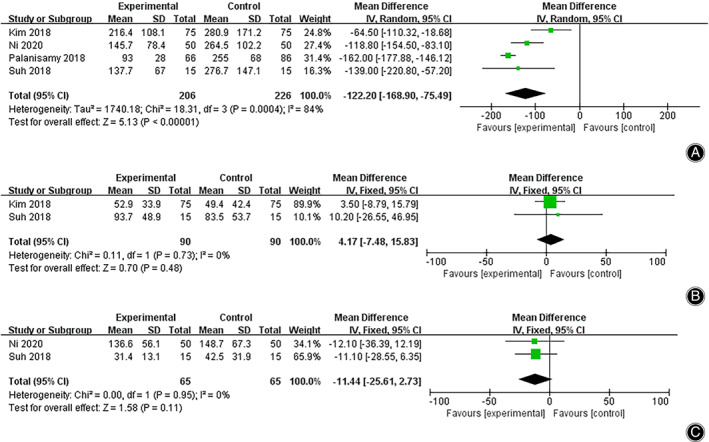
Forest plot of drainage on POD1 (A), POD2 (B), and POD3 (C) after high tibial osteotomy in the TXA and control groups. Abbreviations: POD1, postoperative day 1; POD2, postoperative day 2; POD3, postoperative day 3; TXA, tranexamic acid

Four studies reported the drainage amount on POD1. A random‐effects model was applied (*I* ^2^ = 84%, *P <* 0.0001 in Q statistics),and no significance was found using meta‐regression analysis (total number of patients: *P =* 0.827, age: *P =* 0.553, BMI: *P =* 0.898, preoperative hemoglobin value: *P =* 0.054, M/F ratio: *P =* 0.236). Analysis of the overall effect indicated significantly less drainage on POD1 in the TXA group than in the NTXA group (WMD = –122.20, 95% CI [−168.90, −75.49], *P <* 0.0001). The heterogeneity of the included studies for this outcome should be noted in assessing the reliability of the effect (Fig. 7A, Fig. S7).

#### Drainage Amount on POD2

The drainage amount on POD2 was reported in two studies (*I* ^2^ = 0%, *P =* 0.73). Using the fixed‐effects model, no significant difference was found in the drainage amount on POD2 between the TXA and NTXA groups (WMD = 4.17, 95% CI [−7.48, 15.83], *P =* 0.48) (Fig. 7B, Fig. S8).

#### Drainage Amount on POD3

The drainage amount on POD3 in two of the included studies (*I* ^2^ = 0%, *P =* 0.95) was analyzed with the fixed‐effects model. The results showed no significant difference in the drainage amount on POD3 between the two groups (WMD = –11.44, 95% CI [−25.61, 2.73], *P =* 0.11) (Fig. 7C, Fig. S9).

#### Wound Complications

**Fig. 8:**
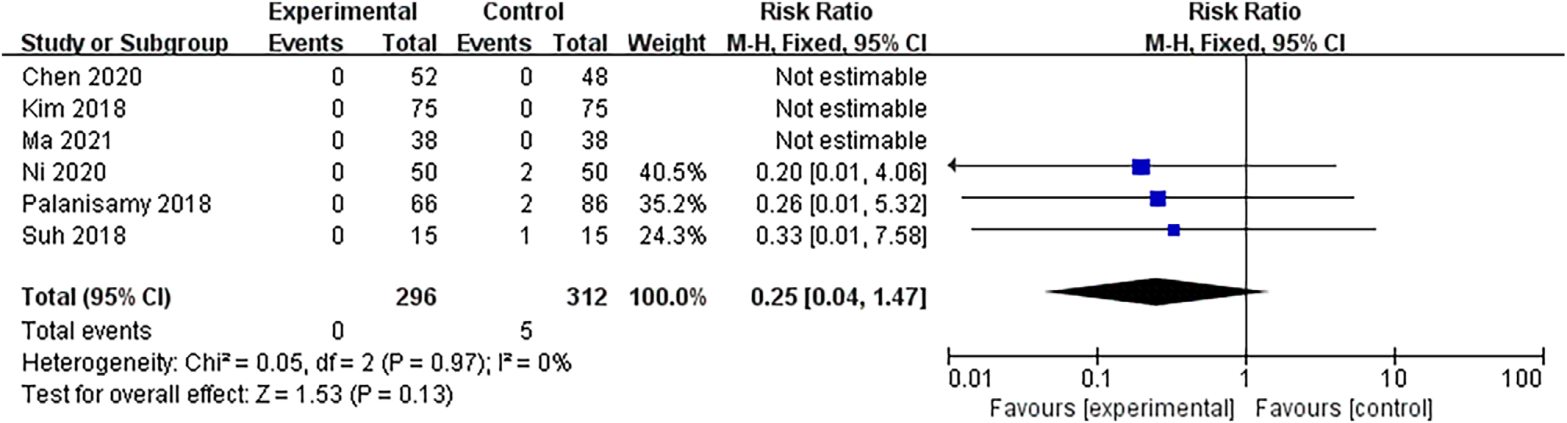
Forest plot of wound complications after a high tibial osteotomy in the TXA and control groups. Abbreviation: TXA, tranexamic acid

Wound complication events including wound infection and hematoma were collected from three studies (*I* ^2^ = 0%, *P =* 0.97 in Q statistics). The fixed‐effects model showed no significant difference in the wound complication rates between the two groups (RR = 0.25, 95% CI [0.04, 1.47], *P =* 0.13) (Fig. 8, Fig. S10).

#### Thromboembolic Events

No new thromboembolic event, including deep vein thrombosis or pulmonary embolism, was reported in any of the included studies.

### Sensitivity Analysis and Publication Bias Assessment

Sensitivity analysis by removing one study at a time showed no changes in the overall or subgroup effect for any of the outcome parameters. Both Egger's test and Begg's test were used to evaluate publication bias. Egger's test could not be applied for outcome parameters reported in only two studies; thus, only Begg's test was performed for these parameters. Potential bias was only detected for transfusion (*P =* 0.025 in Egger's test, *P =* 1.000 in Begg's test); however, trim‐and‐fill analysis suggested that there was no missing data.

## Discussion

To the best of our knowledge, three reviews^19^, ^20^, ^21^ on the effect of TXA during HTO have been published. All reviews reported the efficiency and safety of TXA. However, the number of outcome parameters was limited, and analysis was performed with a high risk of heterogeneity.

### TXA Decreases Total Blood Loss and Hemoglobin Value Changes

In our study, six studies (two RCTs and four cohort studies) with 608 patients were included. We concluded that TXA could significantly reduce blood loss during HTO, which was consistent with previous reports on TXA.^26^, ^27^, ^28^HTO with the opening wedge technique creates a triangle osteotomy gap on the tibial plateau with a correction angle of more than 5°.^29^ In addition, a cut in the bone can lead to excessive blood loss, while TXA can bind to plasminogen competitively to inhibit fibrinolysis, thus reducing blood loss. This effect can be reflected in the hemoglobin value, which is related to the health condition of the patients. A significant effect was also identified on changes in the hemoglobin value on POD1, POD2, and POD5, indicating that the benefit from it can help patients maintain a higher hemoglobin value for a relatively long time. Moreover, patients without intraoperative TXA administration were shown to need more time to recover.

### Females Benefit More from TXA in Terms of Reducing the Total Blood Loss and Total Drainage Amount

High heterogeneity in the total blood loss, total drainage amount, decreased hemoglobin, and drainage amount on POD1 was detected, and meta‐regression analysis was conducted to identify potential risk factors including the total number of patients, age, BMI, preoperative hemoglobin value, and sex ratio. Subgroup analysis based on the sex ratio was performed for the total blood loss and total drainage amount using meta‐regression analysis. The efficiency of TXA on reducing the total blood loss and total drainage amount was demonstrated in each subgroup. The results of subgroup analyses suggested that women might benefit more than men in terms of blood loss management with TXA. Few studies have reported a correlation between sex and TXA use during a HTO. However, a similar correlation has been reported in spinal surgery,^30^, ^31^ arthroplasty replacement,^32^, ^33^, ^34^ and orthognathic surgery.^35^ Hypotheses for the possible mechanisms are as follows. With a relatively low preoperative hemoglobin level, women are thought to have a higher risk of blood loss,^36^, ^37^ and TXA has better effects in patients with higher anticipated blood loss.^38^ A greater muscle mass in men than in women is also hypothesized to cause increased blood loss intraoperatively in women.^30^, ^31^ Sex hormone levels may also play a role, and previous studies have reported their influence on bleeding levels by protecting the vascular integrity.^35^, ^39^ Furthermore, the BMI has been reported to influence the effect of TXA on blood loss.^40^ However, a correlation between the BMI and the effect of TXA on blood loss was not found in our study.

### An Additional Postoperative Dose of TXA Is Suggested to Enhance the Effect

TXA significantly decreased the drainage amount on POD1 but not on POD2 or POD3. In addition, the strongest effect from intraoperative TXA use has been reported to occur during the first 24 h after surgery.^41^ This result may be explained by the 2‐h half‐life of TXA.^42^ As drainage can be a reflection of postoperative blood loss, the significant difference in the drainage amount on POD1 compared to POD2 and POD3 may be in accordance with previous reports, and an extra dose of TXA after POD1 should be considered to enhance the effect.

### No Extra Benefit from TXA on Transfusion and Wound Complications

No significant differences in transfusion or wound complications were detected. Four patients with postoperative transfusions and five with wound complications in the NTXA group were reported compared to none in the TXA group. The transfusion rate is reported to be greater than 30% in knee arthroplasty^43^, ^44^ and greater than 20% in hip arthroplasty without TXA;^45^, ^46^ thus, the effect of TXA on decreasing the transfusion rate in arthroplasty was significant. However, in our study, the transfusion rate was 1.3% in NTXA group. Despite the lack of statistical significance, there may be clinical differences in the transfusion and wound complication rates in clinical practice.

### No New Thromboembolic Event Occurred from TXA Use

Thromboembolic events including deep vein thrombosis and pulmonary embolism might be promoted due to inhibition of fibrinolysis by TXA. In our analysis, none of the six studies reported thromboembolic events, which is consistent with previous studies on the safety of TXA.^47^, ^48^ With an optimum dose, TXA inhibited fibrinolysis only at the surgical site instead of throughout the whole body.

### Limitations

Our study had some limitations that need to be addressed. It included a relatively small number of studies, with two RCTs and four cohort studies. Although subgroup analysis was performed for outcome parameters with high heterogeneity, some subgroups only included one study. The route of administration may also contribute to heterogeneity; however, subgroups were difficult to define because different doses were used in each study. In addition, to minimize potential bias, quality assessment, sensitivity analysis, publication bias tests, and trim‐filling analysis were performed. With regard to the results of total blood loss, group 2 (40% ≥ M/F ≥ 20%) exhibited significantly better effects than group 3 (M/F < 20%); these findings were in contrast with previous evidence and theories. We speculated that this may be a result of the limited number of included studies. Finally, only Asian studies were included, of which three were from China and three were from South Korea.

### Conclusion

TXA is efficient and safe for blood loss management during a HTO. Sex may be an influential factor for the effect, and the benefit of TXA use may last for a long time despite the short half‐life of the drug. More studies are needed to confirm the efficiency, safety, and influential factors.

## Author Contribution

QF contributed to the study design, interpretation of data, and drafting the manuscript; ZZ contributed to the study design, analysis of data, and interpretation of data; DW contributed to data acquisition, analysis of data, and interpretation of data; LMW contributed to data acquisition, analysis of data, and interpretation of data; WX contributed to data acquisition, analysis of data, and interpretation of data; YFT contributed to data acquisition, analysis of data, and interpretation of data; WZL contributed to data acquisition, analysis of data, and interpretation of data; GLW contributed to study design, revising the manuscript, and supervision of the study.

## Supporting information


**Appendix S1** Supporting information

**Appendix S2** Supporting information
