# Supplementary material for: Effect of Tranexamic Acid on Blood Management during a High Tibial Osteotomy: A Systematic Review and Meta‐analysis
Source: Orthop Surg. 2022 Aug 1;14(9):1940–50. doi: 10.1111/os.13407 (PMC9483069; doi:10.1111/os.13407)
Supplement: Supplementary file 1 — Appendix S1 Supporting information [file OS-14-1940-s002.docx]

**Search strategy in Medline and Cochrane Central Register of Controlled Trials**

#1 ((((((((((((Tranexamic Acid[Title/Abstract]) OR (AMCHA[Title/Abstract])) OR (trans-4-(Aminomethyl)cyclohexanecarboxylic Acid[Title/Abstract])) OR (t-AMCHA[Title/Abstract])) OR (AMCA[Title/Abstract])) OR (Anvitoff[Title/Abstract])) OR (Cyklokapron[Title/Abstract])) OR (Ugurol[Title/Abstract])) OR (KABI 2161[Title/Abstract])) OR (Spotof[Title/Abstract])) OR (Transamin[Title/Abstract])) OR (Amchafibrin[Title/Abstract])) OR (Exacyl[Title/Abstract])

#2 ((((high tibial osteotomy[Title/Abstract]) OR (high tibial osteotomies[Title/Abstract])) OR (HTO[Title/Abstract])) OR (tibial surgery[Title/Abstract])) OR (tibial surgeries[Title/Abstract])

#3 #1 AND #2

**Search strategy in Embase**

#1 (Tranexamic Acid or 4 amino methylcyclohexane carboxylate or 4 aminomethylcyclohexanecarbonic acid or 4 aminomethylcyclohexanecarboxylic acid or amca or AMCHA or amchafibrin or amikapron or aminomethyl cyclohexane carboxylic acid or aminomethyl cyclohexanecarboxylic acid or aminomethylcyclohexane carbonic acid or aminomethylcyclohexane carboxylic acid or aminomethylcyclohexanecarbonic acid or aminomethylcyclohexanecarboxylic acid or aminomethylcyclohexanocarboxylic acid or aminomethylcyclohexanoic acid or amstat or anexan or antivoff or anvitoff or caprilon or cis 4 aminomethylcyclohexanecarboxylic acid or cis aminomethyl cyclohexanecarboxylic acid or cl 65336 or cl65336 or cyclocapron or cyclokapron or cyklocapron or cyklokapron or exacyl or fibrinon or frenolyse or hemostan or hexacapron or hexakapron or kalnex or lysteda or micranex or para aminomethylcyclohexane carboxylic acid or rikaparin or ronex or theranex or tramic or tranex or tranexam or tranexanic acid or tranexic or trans 1 aminomethylcyclohexane 4 carboxylic acid or trans achma or trans amcha or trans aminomethyl cyclohexane carboxylic acid or trans aminomethylcyclohexane carboxylic acid or trans aminomethylcyclohexanecarboxylic acid or transamin or transaminomethylcyclohexane carboxylic acid or transexamic acid or traxamic or trenaxin or ugurol).ab. 7466

#2 (high tibial osteotomy or high tibial osteotomies or HTO or tibial surgeries or tibial surgery).ab. 2700

#3 #1 AND #2
