# Supplementary material for: Effect of Tranexamic Acid on Blood Management during a High Tibial Osteotomy: A Systematic Review and Meta‐analysis
Source: Orthop Surg. 2022 Aug 1;14(9):1940–50. doi: 10.1111/os.13407 (PMC9483069; doi:10.1111/os.13407)
Supplement: Supplementary file 2 — Appendix S2 Supporting information [file OS-14-1940-s001.docx]

**Funnel plots for all parameters**


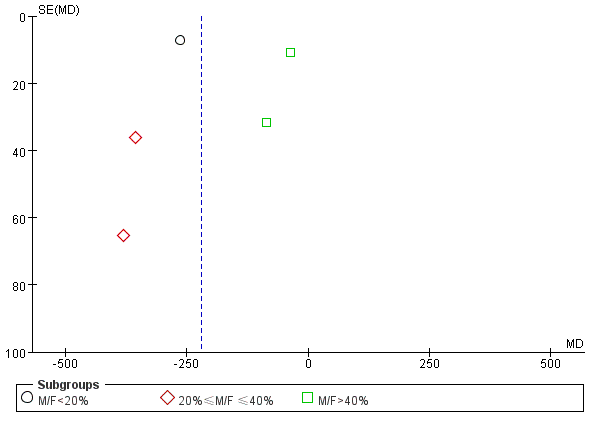


**Figure S1. Funnel plot for total blood loss**


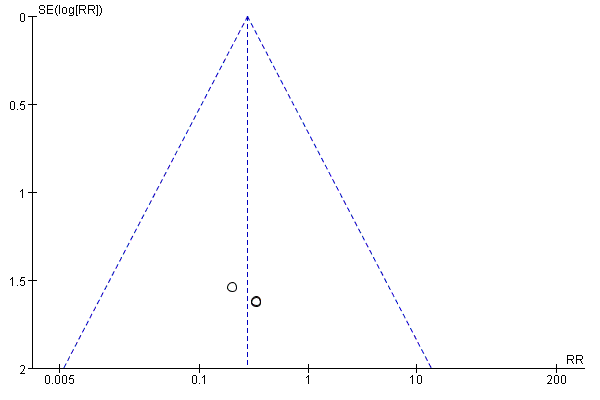


**Figure S2. Funnel plot for transfusion**


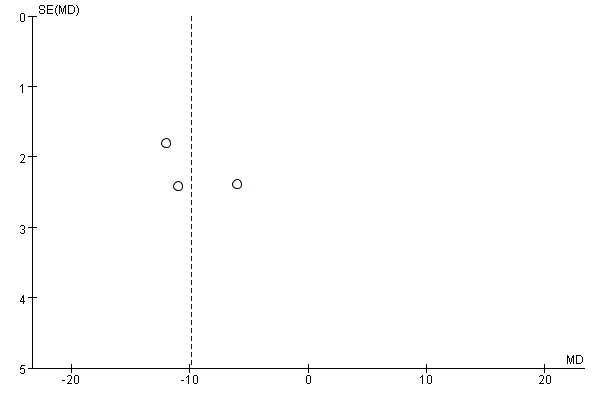


**Figure S3. Funnel plot of hemoglobin decrease on POD1**


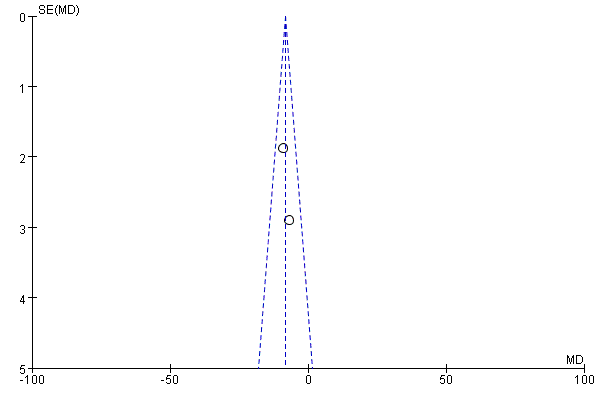


**Figure S4. Funnel plot of hemoglobin decrease on POD2**


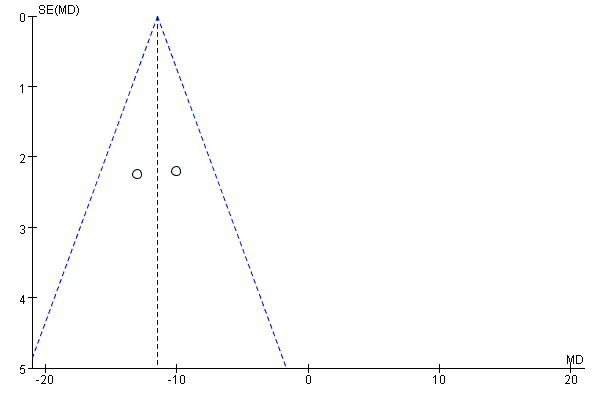


**Figure S5. Funnel plot of hemoglobin decrease on POD5**


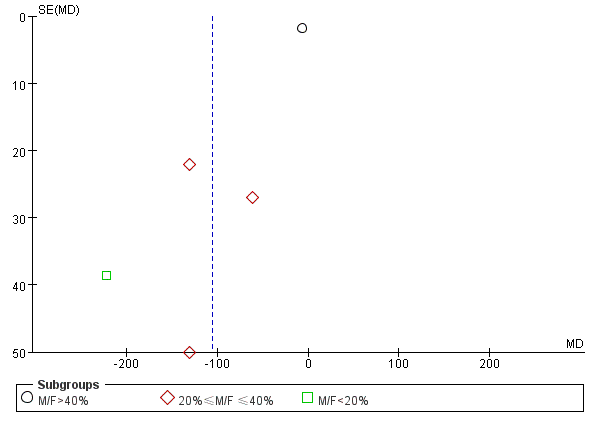


**Figure S6. Funnel plot of total drainage amount**


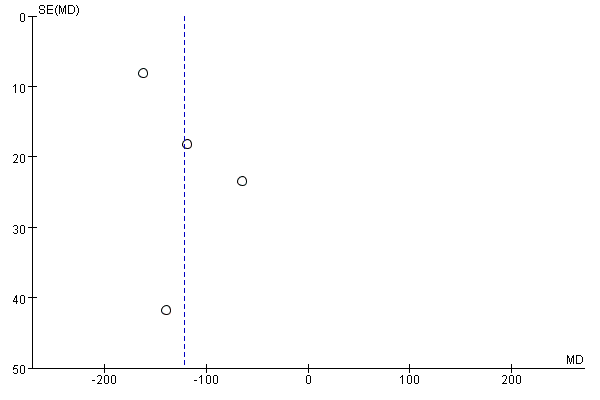


**Figure S7. Funnel plot of drainage amount on POD1**


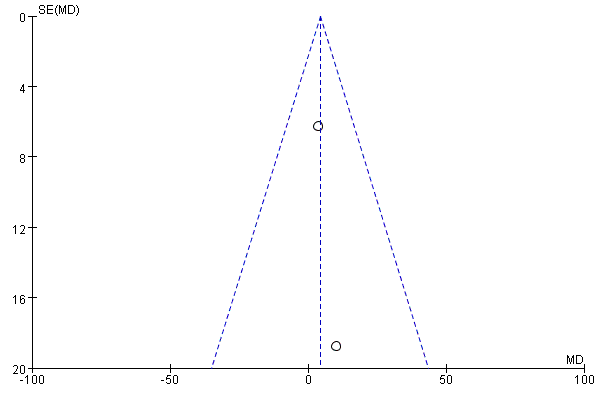


**Figure S8. Funnel plot of drainage amount on POD2**


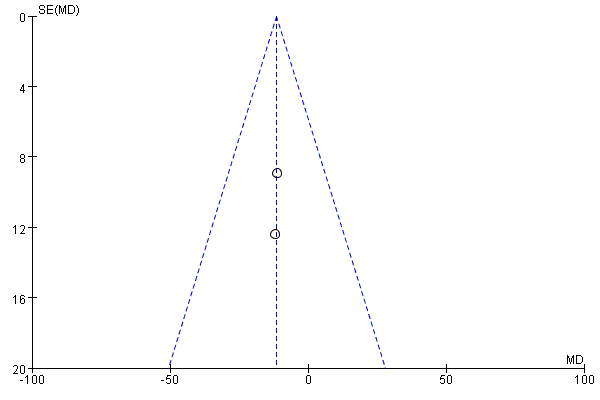


**Figure S9. Funnel plot of drainage amount on POD3**


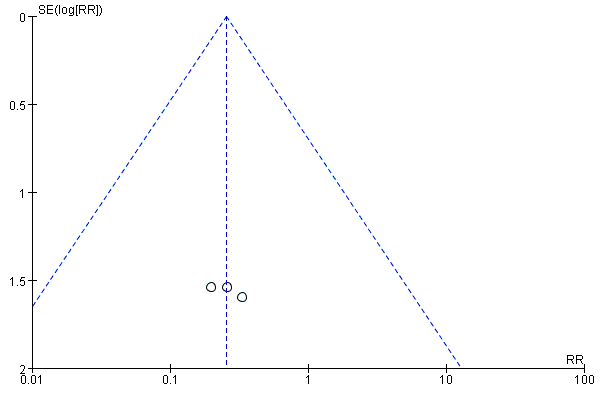


**Figure S10. Funnel plot of wound complications**
